# Multi-sectoral collaborations in selected countries of the Eastern Mediterranean region: assessment, enablers and missed opportunities from the COVID-19 pandemic response

**DOI:** 10.1186/s12961-023-01098-z

**Published:** 2024-01-24

**Authors:** Fadi El-Jardali, Racha Fadlallah, Najla Daher

**Affiliations:** 1 Department of Health Management and Policy, Faculty of Health Sciences, American University of Beirut, Beirut, Lebanon; 2 Knowledge to Policy (K2P) Center, American University of Beirut, Beirut, Lebanon; 3 Department of Health Research Methods, Evidence, and Impact (HEI), McMaster University, Hamilton, ON Canada

**Keywords:** Multi-sectoral collaboration, Multi-sectoral, Inter-sectoral, Collaboration, Coordination, Pandemic, Public health crisis, COVID-19, Eastern Mediterranean region

## Abstract

**Background:**

The coronavirus disease 2019 (COVID-19) pandemic has emphasized the importance of multi-sectoral collaboration to respond effectively to public health emergencies. This study aims to generate evidence on the extent to which multi-sectoral collaborations have been employed in the macro-level responses to the COVID-19 pandemic in nine selected countries of the Eastern Mediterranean region (EMR).

**Methods:**

The study employed in-depth analytical research design and was conducted in two phases. In the first phase, data were collected using a comprehensive documentation review. In the second phase, key informant interviews were conducted to validate findings from the first phase and gain additional insights into key barriers and facilitators. We analysed the macro-level pandemic responses across the following seven components of the analytical framework for multi-sectoral collaborations: (1) context and trigger; (2) leadership, institutional mechanisms and processes; (3) actors; (4) administration, funding and evaluation; (5) degree of multi-sectoral engagement; (6) impact; and (7) enabling factors.

**Results:**

Governments in the EMR have responded differently to the pandemic, with variations in reaction speed and strictness of implementation. While inter-ministerial committees were identified as the primary mechanism through which multi-sectoral action was established and implemented in the selected countries, there was a lack of clarity on how they functioned, particularly regarding the closeness of the cooperation and the working methods. Coordination structures lacked a clear mandate, joint costed action plan, sufficient resources and regular reporting on commitments. Furthermore, there was no evidence of robust communication planning both internally, focused on promoting internal consensual decision-making and managing power dynamics, and externally, concerning communication with the public. Across the selected countries, there was strong representation of different ministries in the pandemic response. Conversely, the contribution of non-state actors, including non-governmental organizations, civil society organizations, the private sector, the media and citizens, was relatively modest. Their involvement was more ad hoc, fragmented and largely self-initiated, particularly within the selected middle- and low income- countries of the EMR. Moreover, none of the countries incorporated explicit accountability framework or included anti-corruption and counter-fraud measures as integral components of their multi-sectoral plans and coordination mechanisms. Key enablers for the adoption of multi-sectoral collaborations have been identified, paving the way for more efficient responses in the future.

**Discussion:**

Mirroring global efforts, this study demonstrates that the selected countries in the EMR are making efforts to integrate multi-sectoral action into their pandemic responses. Nevertheless, persistent challenges and gaps remain, presenting untapped opportunities that governments can leverage to enhance the efficiency of future public health emergency responses.

## Introduction

The coronavirus disease 2019 (COVID-19) pandemic has challenged health systems and economies around the world; a public health crisis of such magnitude revealed that response measures cannot be taken by the health sector alone. The scope and complexity of the pandemic proved that no single agency can work alone to effectively control and mitigate its impact. Therefore, an effective response requires concerted multi-sectoral efforts that involve public, private and civil society actors within and beyond the health sector [1, 2]. In response, countries around the world have taken unprecedented measures to combat the spread of the COVID-19 pandemic while ameliorating its devastating impact on health, society and the economy.

It has been extensively discussed in the literature that tackling the multi-sectoral nature of health challenges requires structured multi-sectoral coordination including state and non-state actors, all of which are critical for shaping a more effective pandemic response, especially a pandemic of the magnitude of COVID-19. The approach of multi-sectoral action for health is widely recognized, with many national and international examples of multi-sectoral approach applications before the COVID-19 pandemic, such as for malaria elimination, tobacco control, human immunodeficiency virus/acquired immunodeficiency syndrome (HIV/AIDS) prevention, Finland’s community‑based cardiovascular disease prevention project (North Karelia Project) and Singapore’s Health Promotion Board [3, 4]. However, successful initiatives in this area remain a challenge for countries worldwide, especially during such public health threats and in vulnerable settings.

The Eastern Mediterranean region (EMR) is distinctly affected by ongoing conflicts, political unrest and displacements. This has posed unique vulnerabilities during the COVID-19 pandemic not experienced in other parts of the world. Despite its unique characteristics, there is still a paucity of information concerning the patterns of multi-sectoral collaborations that exist in this region. It remains unclear to what extent multi-sectoral policies and programs have been adopted in the COVID-19 pandemic responses, including the mechanisms and governance arrangements in place to promote multi-sectoral policies and programs and the key sectors and actors involved.

This study aims to fill the aforementioned gaps by examining the extent to which multi-sectoral collaborations have been employed in the macro-level responses to the COVID-19 pandemic in selected countries of the EMR. The specific objectives are to [5] assess the extent to which a ‘multi-sectoral’ element was employed in the COVID-19 pandemic response in selected EMR countries and [6] explore enablers (i.e. barriers and facilitators) to multi-sectoral collaborations in responding to the COVID-19 pandemic. The significance of this study stems from its examination of whether multi-sectoral collaboration was an opportunity or missed opportunity for a more effective pandemic response in the EMR, identification of key gaps and generation of recommendations on how to promote multi-sectoral collaborations for future public health emergency responses in the EMR and beyond.

### Analytical framework

A multi-sectoral approach refers to deliberate collaboration among various stakeholder groups (e.g. government, civil society and private sector) and sectors (e.g., health, environment and economy) to jointly achieve a policy outcome [3]. While the multi-sectoral approach is advocated as one of the strategies to address complex health and development challenges, there is limited clarity about the process and execution of multi-sector collaboration in practice [7].

To formalize multi-sectoral collaboration for this study, we constructed an analytical framework, building on a suite of existing frameworks on multi-sectoral approaches and action for health, policy analysis and social development [7–13] and drawing from the literature on policy responses to the COVID-19 pandemic [1, 14–17]. The framework encompasses key components and elements critical in a process of multi-sector collaboration (Table 1).

**Table 1:** Analytical framework for multi-sectoral approach to pandemic response

Categories	Elements	Indicators
Context and triggers	Political, social, economic and health contexts driving multi-sectoral collaboration on a particular issue	• Healthcare system • Political structure • Trigger (COVID-19 situation)
Leadership, institutional mechanisms and processes	Leadership structure	• Monarchy versus democracy • Unified versus fragmented governance structures
	Coordination mechanisms	• Mechanisms for coordination, for example, inter-ministerial committees, cabinet committee chaired by the prime minister, etc.) • Mechanism linked to mandate (law, decree, etc.) • Processes for conflict management and building trust
	Communication structures	• A communication plan including strategies to manage power dynamics in conversations • Means of engagement of other sectors, such as consultations, workshops or meetings • External communication with public (including infodemics)
Actors	Sectors & stakeholders	• Leadership in charge of response • Government sectors involved • Non-state actors involved: - Multilateral organizations and United Nations agencies - Local nongovernmental organizations - Public health disciplines/experts - Civil society organizations - Private entities of citizens and media
	Roles and responsibilities	• Roles and responsibilities linked to mandate of collaborative • Written agreement on roles and responsibilities • Standard operating procedures (SOPs) defines rules and norms governing interactions between members
Administration, funding and evaluation	Administration	Level of implementation: • Centralized versus decentralized approach • Community engagement
	Funding	• Amounts and sources of funding for implementation of multi-sectoral response • Cost-sharing mechanism/funding arrangements
	Monitoring and evaluation	• Mechanisms for monitoring and evaluation • Measurable outcomes or indicators set to measure the impact • Accountability framework
Degree of multi-sectoral engagement	Spectrum of engagement	• **Communication:** A one-way relationship in which information from one sector is shared with other sectors • **Cooperation:** This involves optimizing resources while establishing formalities in the work relationships. It results in a loss of autonomy for each sector • **Coordination:** Adjusting the policies and programs of each sector. This leads to increased horizontal networking among sectors. Shared financing sources may be used • **Integration:** This may entail systematic integration of objectives and administrative processes and the sharing of resources, responsibilities and actions. A formal partnership with shared responsibilities ensures achievement of a common goal
Impact	Comprehensiveness of policy measures and responses	• Public health measures • Socio-economic measures • Overall pandemic response
	COVID-19-related outcomes	• Total cumulative COVID-19 cases • % of COVID-19 cases • Total COVID-19 deaths • Case fatality rate (% of deaths out of total cases) • Total tests conducted • Tests/1 million population • % of positive COVID-19 cases out of tests conducted • % of population fully vaccinated
Enabling factors	Barriers and facilitators	• Barriers to multi-sectoral collaboration • Facilitators of multi-sectoral collaboration

This framework was used to analyse the macro-level pandemic response plans in selected EMR countries. For each component, we assessed the extent to which the desirable elements were present and the variations within and across the selected countries.

## Methodology

The study employed an in-depth analytical research design and was conducted in two phases. In the first phase, data were collected using a comprehensive documentation review. In the second phase, key informant interviews were conducted to validate findings from the first phase and gain additional insights and feedback.

The WHO’s classification of the EMR countries was adopted, which includes: Afghanistan, Bahrain, Djibouti, Egypt, the Islamic Republic of Iran, Iraq, Jordan, Kuwait, Lebanon, Libya, Morocco, Oman, Pakistan, the Occupied Palestinian Territories, Qatar, Saudi Arabia, Somalia, South Sudan, Sudan, Syrian Arab Republic, Tunisia, the United Arab Emirates and Yemen [18]. For the purpose of this study, we selected the following nine EMR countries:

- High-income countries (group 1 countries): Bahrain, Saudi Arabia, United Arab Emirates
- Middle-income countries (group 2 countries): Jordan, Lebanon, Tunisia
- Low-income/fragile countries (group 3 countries): Sudan, Syria, Yemen

The nine selected countries represent different income groups (i.e. high-income, middle-income and low-income countries, according to World Bank categorization 2021). They also reflect the three different groupings of EMR countries by the WHO (i.e. group 1, group 2 and group 3 countries) on the basis of population health outcomes, health system performance and level of health expenditure [19].

### Data collection

The data collection process for this study employed a mix of document review and key informant interviews.

#### Document review

This step involved a review of policy documents, protocols, guidelines, strategic plans and programs, legislative acts, national-level reports and research papers to collect data on the different components of the framework. Documents were identified and obtained from websites of governmental bodies, ministries, departments and public agencies of each country. Additionally, we searched the websites of key intergovernmental organizations (e.g. UNDP, UNHCR, WHO, ICRC, UN, World Bank), think tanks, research institutions and universities for relevant reports and studies. We also reviewed key media outlets for relevant news articles on the pandemic response from each selected country.

PubMed and Google Scholar were also searched to retrieve scholarly and peer-reviewed articles using the following search strategy for each country: (“novel coronavirus” OR COVID-19 OR pandemic OR SARS-COV-2) AND (response OR government OR intervention* OR policy OR policies OR national OR “cross-sectoral” OR program* OR intersectoral OR “multi-sectoral” OR governance OR collaboration* OR coordination OR “whole-of-government”) AND ([country name]).

The data collection through document review spanned from January 2020, when the first COVID-19 case was detected in one of the nine countries, to May 2023, when the WHO declared the end of COVID-19 as a global health emergency.

Retrieved documents were reviewed and findings pertaining to the component (and elements) of the framework were abstracted and summarized in a unified excel sheet.

#### Key informant interviews

Key informant interviews were conducted with selected experts/stakeholders from the included countries. The interviews served two purposes: to validate the findings emerging from the documentation review (and fill gaps identified); and to gain additional insights on key barriers, facilitators and lessons learned from promoting multi-sectoral collaboration in the COVID-19 pandemic response. An interview guide was developed, informed by the conceptual frameworks.

We adopted the following stakeholder sampling framework to ensure the selection of right mix of participants for the study [20]:

- Senior- and middle-level policymakers from the public sector;
- Representatives from professional associations who are active in trying to shape and influence health policies
- Researchers who are active in the realm of health systems research and/representatives from university departments and faculties that produce public health and health systems research;
- Representatives of the non-state sector who are active in trying to shape and influence health policies.

We purposively mapped stakeholders from the selected countries against the framework. We targeted those stakeholders who participated in the COVID-19 policy process, including policymakers involved in national pandemic policies and response plans; NGOs involved in COVID-19 programs; and researchers/academia informing the COVID-19 response. A total of 24 stakeholders were initially selected and invited to participate in the study. Following two reminder emails, eight participants (three policymakers, two researchers, one public health expert, one representative from EMR Public Health Network and one senior representative from an intergovernmental organization) agreed to take part in the study. Participants represented the following low- and middle-income countries: Jordan, Lebanon, Sudan, Syria, Tunisia and Yemen.

The meetings were conducted virtually (on Zoom) by the lead researcher (F.E.J.) or co-lead researchers (R.F., N.D.) and lasted between 40 min and 60 min each. Responses were documented through note-taking by the researchers (R.F., F.E.J., N.D.).

The key informant interviews were conducted between October 2020 and March 2021.

### Data analysis and synthesis

The data generated from the documentation review and key informant interviews were collated and analysed in aggregate form and categorized according to the seven components of the analytical framework (Table 1); meaning that findings were analysed according to components of the framework rather than by source of data. We used a deductive content analysis approach to synthesize data, which is appropriate for policy-relevant qualitative data [9, 21]. This approach uses an analytical framework featuring key constructs and variables as initial coding categories while leaving room for other emerging themes outside the framework.

The first stage of data analysis comprised analysing and coding data obtained from the documentation review according to each of the seven components of our set framework. Once the findings from the documentation review were coded, the interviews with stakeholders were conducted. The purpose of the interviews was to validate the findings generated from the first step of analysis and obtain additional insights about each component of the analytical framework. Findings from interviews were also coded according to the analytical framework adopted for this study. Emerging themes were compared with those from the documentation review, and information was added or validated where appropriate.

Data triangulation helped provide a more in-depth understanding of the issue and increase the reliability and validity of findings through cross-checking of information across different data sources.

The study was conducted following standard ethical guidelines and protocols. Participation in this study was voluntary. A verbal consent form was provided to participants prior to commencing with the interviews. The confidentiality and anonymity of responses were ensured at all times. No names or identifiers were linked to any of the findings emerging from the study.

## Results

Findings are presented according to the components described in the analytical framework: (1) context and trigger; (2) leadership, institutional mechanisms and processes; (3) actors; (4) administration, funding and evaluation; (5) degree of multi-sectoral engagement; (6) impact; and (7) enabling factors.

### Context and trigger

#### Healthcare system

The EMR represents a diverse landscape of healthcare systems and political structures, each shaped by unique historical, socio-economic and political factors (Table 2). Among the nine selected EMR countries, high-income countries such as Bahrain, Saudi Arabia and the United Arab Emirates possess robust healthcare systems underpinned by strong financial backing and effective governance [22–24]. In contrast, countries such as Lebanon, Tunisia and Jordan face a multitude of challenges, including political instability, economic uncertainties, a high influx of refugees and relatively less efficient healthcare systems [25–27]. Conflict-affected countries such as Sudan, Syria and Yemen, already burdened by other disease outbreaks, famine and malnutrition, relied heavily on humanitarian assistance from donor nations and non-governmental organizations for resources [28, 29].

**Table 2:** Health systems, political structures and COVID-19 situation in selected countries

Country	Health system characteristics	Political structure and health policymaking process	COVID-19 situation during early phase of pandemic (as of 31 December 2020)	References
*High-income countries*				
Bahrain	• **Governance arrangement:** The National Health Regulatory Authority (NHRA) oversees Bahrain’s healthcare system, regulating both public and private providers • **Financing arrangement: **Health expenditure as a share of GDP reached 4.7% in 2020. The Ministry of Health provides free healthcare to Bahrainis while private hospitals offer paid services. Out-of-pocket expenses account for only 20% of total health expenditures, indicating a strong safety net for the population • **Service delivery:** Multiple entities deliver healthcare services, with the Ministry of Health providing the bulk of subsidized care. A mix of public and private healthcare providers caters to the needs of the population	• **Government type:** Constitutional monarchy • **Health policymaking:** Healthcare development is guided by several mid- and long-term strategies and policy documents, including the National Health Plan 2016–25, adopted by the Supreme Council of Health; the National Health Regulatory Authority 2016–20 Strategy, emphasizing inspection and accreditation activities; and the Bahrain Economic Vision 2030, a long-term economic development and diversification agenda that seeks to modernize Bahrain’s healthcare sector	• **Date of first recorded COVID-19 case**: 24 February 2020 • **% of COVID-19 cases out of the population**: 5.34% • **Total COVID-19 deaths**: 352 • **Case fatality rate** (% of deaths of total cases): 0.38%	[23, 30–32]
Kingdom of Saudi Arabia (KSA)	• **Governance arrangement:** Government has given high priority to the development of healthcare services which resulted in a well-established healthcare system that provides all citizens and residents with free healthcare services • **Financing arrangement:** Government is the primary funder of the healthcare system and has significantly increased its spending on the healthcare sector over the past few years. In 2020, the government allocated $US 39.2 billion for the health sector • **Service delivery:** The government is responsible for providing healthcare services. As of 2021, there were more than 75,000 hospital beds, which translates to 2.3 beds per 1000 people	• **Government type:** Monarchy, headed by the king, who is also the commander-in-chief of the military • **Health policymaking:** Health policymaking navigates a dynamic landscape, balancing adherence to global patient safety standards and international best practices with statutory regulations, cultural sensitivities and alignment with national health development strategies	• **Date of first recorded COVID-19 case**: • 2 March 2020 • **% of COVID-19 cases out of the population**: 1.03% • **Total COVID-19 deaths**: 6223 • **Case fatality rate** (% of deaths of total cases): 1.71%	[5, 24, 33–36]
United Arab Emirates (UAE)	• **Governance arrangement:** A comprehensive, government-funded health service and a rapidly developing private health sector that delivers a high standard of healthcare to the population. Healthcare is regulated at both the federal and emirate level. Public healthcare services are administered by different regulatory authorities in the UAE • **Financing arrangement:** Government allocates a significant share of the federal budget for the healthcare sector every year to provide quality medical services. The percentage of federal budget allocated to health was 7.3% in 2019 • **Service delivery:** Well-functioning healthcare system at primary, secondary and tertiary levels. The UAE has a top-tier healthcare system that is regularly ranked in the top 20 in the world with adequate resources including financial and well-developed health system infrastructure	• **Government type:** Elective monarchy formed from a federation of seven emirates. Each emirate is an absolute monarchy governed by a ruler, and together the rulers form the Federal Supreme Council • **Health policymaking:** The UAE’s public policy for healthcare focuses on developing organizational and legal frameworks based on best practices, and overhauling and upgrading the private and public sector health service capabilities. In addition, public policy action will set priorities for health services development within the sector	• **Date of first recorded COVID-19 case**: 29 January 2020 • **% of COVID-19 cases out of the population**: 2.08% • **Total COVID-19 deaths**: 669 • **Case fatality rate** (% of deaths of total cases): 0.32%	[22, 37–40]
*Middle-income countries*				
Jordan	• **Governance arrangement:** The Jordanian health system is governed by the Higher Health Council, which sets national health policy, regulates the sector and plans for equitable and high-quality services • **Financing arrangement:** Health expenditure in Jordan represents 8.44% of GDP. Public, military, private and university health insurance schemes cover the majority of the population • **Service delivery:** Jordan’s health system is a complex amalgam of three major sectors: public, private and donors. Key challenges include accessibility issues, service duplication, poor coordination and unregulated private sector, low private sector utilization, limited quality improvement programs, inefficient resource usage, poor management and an inadequate health information system	• **Government type:** A monarchy in which the king plays a dominant role in politics and governance. The parliament’s lower house is elected • **Health policymaking:** Health policymaking in Jordan is highly centralized, and local governments have no autonomy to formulate their own responses to the pandemic	• **Date of first recorded COVID-19 case:** 2 March 2020 • **% of COVID-19 cases out of the population**: 2.87% • **Total COVID-19 deaths**: 3834 • **Case fatality rate** (% of deaths of total cases): 1.3%	[41–44]
Lebanon	• **Governance arrangement**: Lebanon’s healthcare system is pluralistic and fragmented, private sector dominated, and curative focused. Existing structural challenges, economic crisis, civil unrest and Beirut Port Blast have propelled the system towards collapse • **Financing arrangement:** Private sector is the main financer of the health system. Public healthcare services receive limited resources from the government, particularly for primary and preventive care, while a significant percentage is spent on hospital-based care • **Service delivery:** Healthcare delivery in Lebanon is undertaken by a network of 134 private and 28 public hospitals in addition to various nongovernmental organizations providing primary healthcare in underprivileged regions	• **Government type:** A parliamentary democratic republic with direct parliamentary elections held every 4 years. However, after the 2009 election, no further elections were held until 2018 and then again in 2022 • **Health Policymaking:** Lebanon’s political system, characterized by a corrupt sectarian structure, has hindered healthcare sector development. The absence of political will and inadequate accountability mechanisms have contributed to insufficiencies in the healthcare sector, particularly during the crisis	• **Date of first recorded COVID-19 case:** 21 February 2020 • **% of COVID-19 cases out of the population**: 2.66% • **Total COVID-19 deaths**: 1468 • **Case fatality rate** (% of deaths of total cases): 0.8%	[27, 45–54]
Tunisia	• **Governance arrangement**: The healthcare system is overseen by the Ministry of Health, which is responsible for developing and implementing national health policies, regulating the healthcare sector and planning for equitable and high-quality healthcare services. The Ministry also manages the National Health Insurance Fund (CNAM), which provides health insurance coverage to a significant portion of the population • **Financing arrangement:** Healthcare is financed through a combination of social health insurance, general government revenues and private spending. CNAM covers more than 80% of the population, while the non-contributory Assistance Médicale Gratuite (AMG) program covers poor households. Despite the existence of CNAM and government funding, out-of-pocket payments remain a substantial source of health expenditure, accounting for 36.6% of total health spending • **Service delivery:** Tunisia’s healthcare infrastructure is characterized by uneven distribution and resource disparities. This uneven distribution highlights the coastal–interior regional gap, leaving interior regions more vulnerable	• **Government type:** Tunisia, once a representative democracy, transitioned to an authoritarian state following the president’s suspension of parliament and rule by decree in 2021. The country’s political landscape is fragmented, with a deeply divided parliament hindering effective policymaking • **Health policymaking:** National public health policy is implemented by regional health directorates under the supervision of the Ministry of Public Health. However, the centralized management of public health facilities is reported to hinder responsiveness	• **Date of first recorded COVID-19 case:** 2 March 2020 • **% of COVID-19 cases out of the population**: 1.17% • **Total COVID-19 deaths**: 4676 • **Case fatality rate** (% of deaths of total cases): 3.36%	[55–58]
*Low-income countries*				
Sudan	• **Healthcare system governance**: The Federal Ministry of Health oversees the Sudanese healthcare system in collaboration with other ministries and state health departments. Despite decentralization efforts, the system remains incomplete due to the inappropriate transfer of responsibilities to lower levels, resulting in low implementation efficiency due to weak capacity and resource constraints • **Healthcare system financing:** The former Sudanese government prioritized military spending over healthcare, leaving the current transitional government with a severely underfunded and fragmented health system, receiving only 1% of the budget. This underfunding has led to high out-of-pocket expenditures (66.95%), causing catastrophic financial hardship for 78% of households • **Service delivery:** Sudan’s healthcare infrastructure is severely lacking. When the COVID-19 pandemic began, the country had only around 500 intensive care unit (ICU) beds. Moreover, there were only 1.2 hospitals and 13.5 primary healthcare centres per 100,000 residents. The system is further strained by a shortage of healthcare professionals, inadequate supplies and logistics and severely understaffed and under-equipped health facilities	• **Government type:** Sudan’s political system is characterized by an authoritarian rule with the president and his National Congress Party (NCP) maintaining power through a combination of repression and inducements. This authoritarian structure has significantly impacted the country’s healthcare system and policymaking processes • **Health policymaking:** Health policymaking in Sudan is primarily driven by the Federal Ministry of Health (FMoH), which is responsible for formulating and implementing national health policies. However, the authoritarian nature of the government has limited the participation of other stakeholders, such as civil society organizations and healthcare professionals, in shaping health policies	• **Date of first recorded COVID-19 case:** 13 March 2020 • % of COVID-19 cases out of the population:** 0.0056%** • Total COVID-19 deaths: **1468** • Case fatality rate (% of deaths of total cases):** 5.75%**	[59–62]
Syria	• **Governance arrangement:** Syria’s healthcare system is fragmented due to the ongoing conflict and the absence of a central national authority. Multiple power structures have emerged, each with its own governing body responsible for healthcare provision. These structures vary in their effectiveness and coordination, hindering overall system management • **Financing arrangement:** Syria’s healthcare system has faced significant financial constraints even before the conflict. The limited resources available have been insufficient to meet the growing demand for healthcare services, leading to low government expenditure on health as a percentage of gross domestic product (GDP) and per capita. This shortage of funding has resulted in high out-of-pocket expenses for patients • **Service delivery:** The Syrian healthcare system has been severely impacted by the conflict, with many public hospitals damaged or destroyed. Approximately 40% of public hospitals are either partially or fully functional, severely limiting the capacity to provide essential healthcare services. Additionally, the overall healthcare infrastructure is lacking, with insufficient hospital beds and inadequate supplies. This situation has been exacerbated by the COVID-19 pandemic, which has further strained the already overburdened system	• **Government type:** Syria is a unitary multiparty republic with a one-chamber legislative body, headed by a president. However, the country’s ongoing conflict has fragmented governance structures, leading to the emergence of multiple power centres in different geographical regions. This political division has resulted in a patchwork of health systems, each with varying capacities and structures, affecting the delivery of healthcare services to the Syrian population • **Health policymaking:** The complex and dynamic nature of Syria’s conflict has disrupted traditional healthcare governance structures, leading to a situation in which policymaking is influenced by the shifting power dynamics and territorial control within different regions. This fragmentation has hindered the development and implementation of coherent national health policies, affecting the equitable and efficient delivery of healthcare services to the population	• **Date of first recorded COVID-19 case:** 22 March 2020 • **% of COVID-19 cases out of the population**: 0.064% • **Total COVID-19 deaths**: 5373 • **Case fatality rate** (% of deaths of total cases): 6.2%	[63–65]
Yemen	• **Governance arrangement:** Yemen’s healthcare system is structured at three levels: central, governorate and district. While the central Ministry of Health oversees policy and strategy development, governorate and district health offices manage service delivery, including planning, budgeting and human resource management • **Financing arrangement:** Yemen’s annual total health expenditure is among the lowest globally. Yemen’s healthcare system heavily relies on external funding, with implementing organizations playing a primary role in service delivery. This over-dependence on development partners has exacerbated challenges, as external funding has declined significantly, leaving the system vulnerable to threats such as COVID-19 • **Service delivery:** The ongoing conflict has severely disrupted Yemen’s healthcare system, with only 50% of facilities fully functional. This has led to critical shortages in essential medicines, staff and equipment, compromising access to basic healthcare services for three quarters of the population. The conflict has also exacerbated health challenges and weakened governance in the healthcare sector	• **Government type:** A complex and fragmented governance structure, with no single entity exercising full control over the territory. The central government has been effectively paralysed since the outbreak of the civil war in 2015, and various armed groups and unelected officials hold sway over different regions. This fragmented political landscape has significantly hindered the development and implementation of effective health policies • **Health policymaking**: The fragmented governance structure and weak institutional capacity have severely constrained health policymaking in Yemen. The absence of a central authority capable of coordinating and implementing health policies has resulted in a patchwork of approaches across different regions. Additionally, deficiencies in the health information system hinder evidence-based decision-making and resource allocation, further impeding effective policy formulation	• **Date of first recorded COVID-19 case:** 10 April 2020 • **% of COVID-19 cases out of the population**: 0.007% • **Total COVID-19 deaths**: 610 • **Case fatality rate** (% of deaths of total cases): 29%	[66–71]

#### Political structures

Table 2 provides an overview of the political structure in the nine selected countries. Lebanon stands out as a parliamentary democracy, distinguishing itself from the others. Bahrain, Saudi Arabia, the United Arab Emirates and Jordan operate under a monarchy system. Tunisia experienced a notable shift from a representative democracy to an authoritarian state in 2021. Meanwhile, political conflicts in Sudan, Yemen and Syria have led to a weakened political structure and fragmented governance.

#### Trigger (COVID-19 situation)

The COVID-19 pandemic, with its rapid and pervasive spread, exposed the strengths and weaknesses of healthcare systems across the EMR. The first confirmed case in the region was reported in the UAE on 29 January 2020, marking the beginning of the pandemic’s relentless march across the EMR [72]. Within a short period, all nine selected countries had recorded their first cases, highlighting the urgent need for immediate and effective public health responses (Table 2). The sweeping scale, breadth and impact of the pandemic rendered it of utmost priority. The recognized interdependencies amongst sectors and the need to work with others triggered the establishment of multi-sectoral collaboration in response.

The functioning of multi-sectoral collaboration varied across the region. Saudi Arabia, Bahrain and the United Arab Emirates, characterized by monarchical governments and robust healthcare infrastructure, implemented swift and efficient measures, including widespread testing and early lockdown protocols. Despite its monarchical system, Jordan faced challenges in responding to the pandemic due to limited healthcare preparedness and the strain of hosting a large refugee population [73]. On the contrary, countries with political and economic instability struggled with fragmented and often delayed responses [55, 59, 66]. Lebanon’s private-sector-dominated healthcare system and persistent political turmoil, exacerbated by inadequate accountability mechanisms, contributed to insufficiencies in the healthcare system and impeded a cohesive response, while Tunisia’s political transition from a representative democracy to an authoritarian state affected its healthcare response and resource allocation [26, 27, 46]. In countries with fragmented governance structures and weak healthcare systems such as Sudan, Syria and Yemen, the lack of coordination between government agencies and non-health sectors weakened the pandemic’s impact, particularly in terms of resource allocation and service delivery. These disparities were evident in the early phase of the pandemic, as COVID-19 outcomes diverged significantly across the EMR [74–76]. UAE and Bahrain recorded lower case fatality rates (0.32% and 0.38%, respectively) during the first year of the pandemic. In contrast, Yemen had the highest case fatality rate (29%), followed by Syria (6.2%) and Sudan (5.75%) (Table 2).

### Leadership, institutional mechanisms and processes

#### Leadership role

Since the confirmation of the first COVID-19 cases in the EMR in late January 2020, the nine countries selected for this study have developed national preparedness and response plans as part of the multi-sectoral COVID-19 response. In the majority of the high-income (Bahrain, UAE) and middle-income (Jordan, Tunisia) countries, the multi-sectoral response has been led by the government through the engagement of the prime minister’s offices, high governmental and ministerial councils, or offices in charge of crises of national concern. In contrast, the response in low-income countries (Sudan, Syria, Yemen) has been led by intergovernmental agencies [specifically United Nations (UN) entities and WHO] in collaboration with respective ministries of health, which did not permit significant interactions among other government departments (see Table 3).

**Table 3:** Leadership, institutional mechanisms and processes for multi-sectoral collaboration

Country	Multi-sectoral initiative	Entity that led the response	Coordination mechanism mandated by law	Mechanism to facilitate interaction between sectors/actors	Mechanisms for communication	Mechanism for conflict management and building trust
*High-income countries*						
Bahrain	The National Health Emergency Preparedness and Response plan	His Royal Highness (HRH) the crown prince and prime minister	Not found	The National Taskforce for Combating Coronavirus (COVID-19) chaired by the Prime Minister Activated mid-February 2020; first meeting: 25 February 2020	Regular meetings of the taskforce followed by a press conference and briefing	Not found
Kingdom of Saudi Arabia	The pandemic control plan	Government	Not found	COVID-19 follow-up committee chaired by the Ministry of Health Activated early January 2020; first meeting on 1 February 2020	Regular meetings of the COVID-19 follow-up committee in person	Not found
United Arab Emirates	The UAE Government’s Initiatives to Combat the COVID-19 Crisis	The Supreme Committee of Crisis and Disaster Management	Yes	National Emergency Crisis and Disasters Management Authority (NCEMA) already in place since 2007; first meeting held on 31 October 2020 National COVID-19 Crisis Recovery Management and Governance Committee; first meeting held on 4 November 2020	Regular meetings, the committee presents a weekly report on developments to the cabinet; all governmental authorities were getting guidance and support as well as reporting directly to NCEMA	Not found
*Middle-income countries*						
Jordan	National COVID-19 Preparedness and Response Plan 2020	The government through the National Center for Security and Crisis Management	Yes	Coronavirus Crisis Cell within the existing structure of the National Center for Security and Crisis Management (NCSCM); Activated January 2020	Regular meetings and press briefings	Not found
Lebanon	The COVID-19 national preparedness and response	Government	Yes	National Committee for COVID-19 (NCC) chaired by the ministry of health; Activated 22 January 2020	Regular meetings of the committee followed by recommendations	Not found
Tunisia	Preparation and Response Plan at the risk of introduction and dissemination of “SARS-CoV-2” in Tunisia	Government	Yes	• The National Coronavirus Response Authority supervised by the prime minister • Permanent National Committee for Disaster Prevention and Response and Relief Organization • The National Security Council headed by the president Mechanisms activated between 21 and 25 March 2020	Daily meetings of the Local Crisis Committee members	Not found
*Low-income countries*						
Sudan	COVID-19 Country Preparedness and Response Plan (CPRP)	-Federal Ministry of Health with the support of the WHO (led the response) – ruling cabinet of ministers and the Sovereign Council (formed High Committee for Health Emergencies)	Not found	The High Committee for Health Emergencies (to coordinate governmental and non-governmental efforts for combating COVID-19); Activated early March 2020	Ad hoc meetings as needed	Not found
Syria	National preparedness and response in Syria	The UN Resident Coordinator and Humanitarian Coordinator (RC/HC) with the WHO Representative for Syria	Not found	COVID-19 Crisis Coordination Committee; Activated August 2020	Weekly Health Sector coordination meetings/operational calls/daily WHO meetings	Not found
Yemen	Yemen National COVID-19 Preparedness and Response Plan	WHO (national health cluster to coordinate humanitarian health responses) led the response; the Ministry of Public Health and Population (MOPHP) co-led the response	Not found	Inter-ministerial emergency COVID-19 Response Committee; Emergency Committee in the Southern; Activated 16 March 2020	The Task Force meets on a weekly basis in addition to ad hoc meetings organized as needed	Not found

#### Coordination mechanisms

In the majority of the selected EMR countries, coordination mechanisms were established to facilitate interaction between sectors and actors. Specifically, the formation of coordination committees at the level of the prime minister’s or president’s office, or cross-ministerial committees at the ministry level, were identified as the primary mechanisms through which these forms of multi-sectoral action were realized. However, the mandate and influence of these committees varied, with uncertainties regarding how they functioned, particularly in terms of the closeness of the cooperation and the working methods (Table 3). Coordination structures lacked a clear mandate, joint costed action plan, adequate resources and regular reporting on commitments. Additionally, there were no explicit processes for conflict management and building trust among stakeholders.

#### Communication structures

Beyond bringing sectors together, effective collaboration requires sectors to communicate and engage in meaningful participation, both internally and externally with the public. In the majority of the selected countries, regular meetings were reportedly being held (either in person or online), some of which were followed by press briefings. However, there was no evidence of robust communication planning, including strategies for promoting consensual decision-making and managing power dynamics in conversations (Table 3).

The COVID-19 pandemic also underscored the importance of knowledge generation and utilization in driving effective responses. This process, however, faced significant challenges, such as scientific uncertainties, the spread of misinformation and weak collaborations among stakeholders [77]. Various government initiatives aimed to combat misinformation and disseminate accurate information through hotlines, websites and media collaborations with health experts [78, 79]. Despite these efforts, their effectiveness was often compromised by factors such as limited government capacity, perceived corruption and non-transparent decision-making processes. Particularly, in conflict-affected countries such as Sudan, Syria and Yemen, the national governments’ weak capacity and perceived corruption undermined the legitimacy and trust in pandemic response authorities [28, 29]. Throughout the pandemic, some of these countries were still withholding COVID-19 data, influenced by a lack of political will and underdeveloped digital data collection systems. This "secrecy" and institutionalized opacity eroded public trust and hindered a successful multi-sectoral response.

### Actors

Given the scale and breadth of the COVID-19 pandemic, a whole-of-government, whole-of-society approach was needed to respond effectively. Actors from three critical sectors – public, private and civil society – have been involved in the response of the nine selected countries, albeit to varying degrees; furthermore, roles and responsibilities among actors in the collaborative arrangements have not been clearly defined nor linked to a mandate for a more effective multi-sectoral response.

When it comes to state actors, there was a strong representation of different ministries in the national responses of the nine selected countries. The prime minister’s office was also involved in many cases (Table 4). Conversely, the contribution of non-state actors, including non-governmental organizations (NGOs), civil society organizations (CSOs), the private sector, the media and citizens, was relatively modest in most of the selected countries, with the exception of Sudan. The nature of these initiatives varied depending on the context. In cases in which governments exhibited weakness, such as in Lebanon, non-state actors often took the lead in initiating and carrying out relief efforts [80, 81]. Conversely, in contexts in which governments maintained a strong presence, such as in Bahrain, Saudi Arabia and United Arab Emirates, non-state actors’ involvement was often prompted or initiated by government initiatives [82–85].

**Table 4:** Sectors and actors involved in the COVID-19 pandemic response

Country	Sectors and actors
Bahrain	Ministry of Health; Ministry of Works, Municipal Affairs and Urban Planning; the Bahraini Civil Aviation Affairs; the Bahrain National Task force; the Bahrain Defense Force Royal Medical Services military hospital
Kingdom of Saudi Arabia	Representatives from 13 ministries: Health, Defense, Energy, Interior, National Guard, Foreign Affairs, Finance, Media, Commerce and Investment, Hajj and Umrah, Education and Tourism. Also present was the General Authority of Civil Aviation, the Saudi Red Crescent Authority, the Saudi Food and Drug Authority, the General Authority of Customs and the Saudi Center for Disease Prevention and Control
United Arab Emirates	The Ministry of Presidential Affairs; Ministry of Interior; Ministry of Defense; Ministry of Foreign Affairs and International Cooperation; Ministry of Health and Prevention; Ministry of Economy; Ministry of Finance; Ministry of Education; Ministry of Human Resources and Emiratization; Ministry of Community Development; Ministry of Energy and Infrastructure; Ministry of Industry and Advanced Technology; Ministry of Food and Water Security; Prime Minister’s Office; General Secretariat of the Cabinet; General Secretariat of the Supreme Council for National Security; etc.
Jordan	Ministry of Health, Ministry of Culture, Ministry of Education, Ministry of Industry, Trade and Supply, the Ministry of Social Development, the Ministry of Youth, the Ministry of Communication, the Government Coordinator for Human Rights and the Prime Minister’s office
Lebanon	Ministry of Public Health, the Ministry of Agriculture, the Ministry of Foreign Affairs, the Ministry of Social Affairs, the Ministry of Education and Higher Education, the Ministry of Public Works and Transport, the Ministry of Defense, the Ministry of Interior and Municipalities, the Ministry of Information, and the Disaster Risk Management Unit at the Prime Minister’s Office
Tunisia	Ministry of Interior, Ministry of Finance, Ministry of Planning and Regional Development, Ministry of Agriculture, Ministry of Equipment and Housing, Ministry of Environment and Territorial Development, Ministry of Transport, Ministry of Communications, Ministry of Public Health, Ministry of justice, Ministry of Defense, Ministry of Foreign Affairs and the Head of the National Intelligence Center
Sudan	The Committee is made of members from the Ministries of Health, Labor and Social Welfare, Foreign Affairs, Internal Affairs, Information and Finance, as well as the head of the Central Bank of Sudan and representatives of the army, the police and the security services. The WHO, UN agencies, the ruling cabinet of ministers and the Sovereign Council
Syria	Ministry of Health, Ministry of Education; Ministry of Interior; Higher Education; Local Administration and Environment; Social Affairs and Labor; and Ministry of Foreign Affairs; Idleb Health Directorate; WHO; the United Nations High Commissioner for Refugees (UNHCR); the United Nations International Children’s Emergency Fund (UNICEF); the United Nations Population Fund (UNFPA); the United Nations Development Programme (UNDP); and the United Nations Office for the Coordination of Humanitarian Affairs (UNOCHA). In addition, sectors, including Water, Sanitation and Hygiene (WASH), Health, Logistics, Protection, Nutrition, Food Security, Shelter and Non-Food Items (NFIs)
Yemen	WHO, Ministry of Public Health and Population, Ministry of Foreign Affairs, Ministry of Education and Higher Education, Ministry of Public Works and Transport, Ministry of Interior and Municipalities and Ministry of Information

**Fig. 1:**
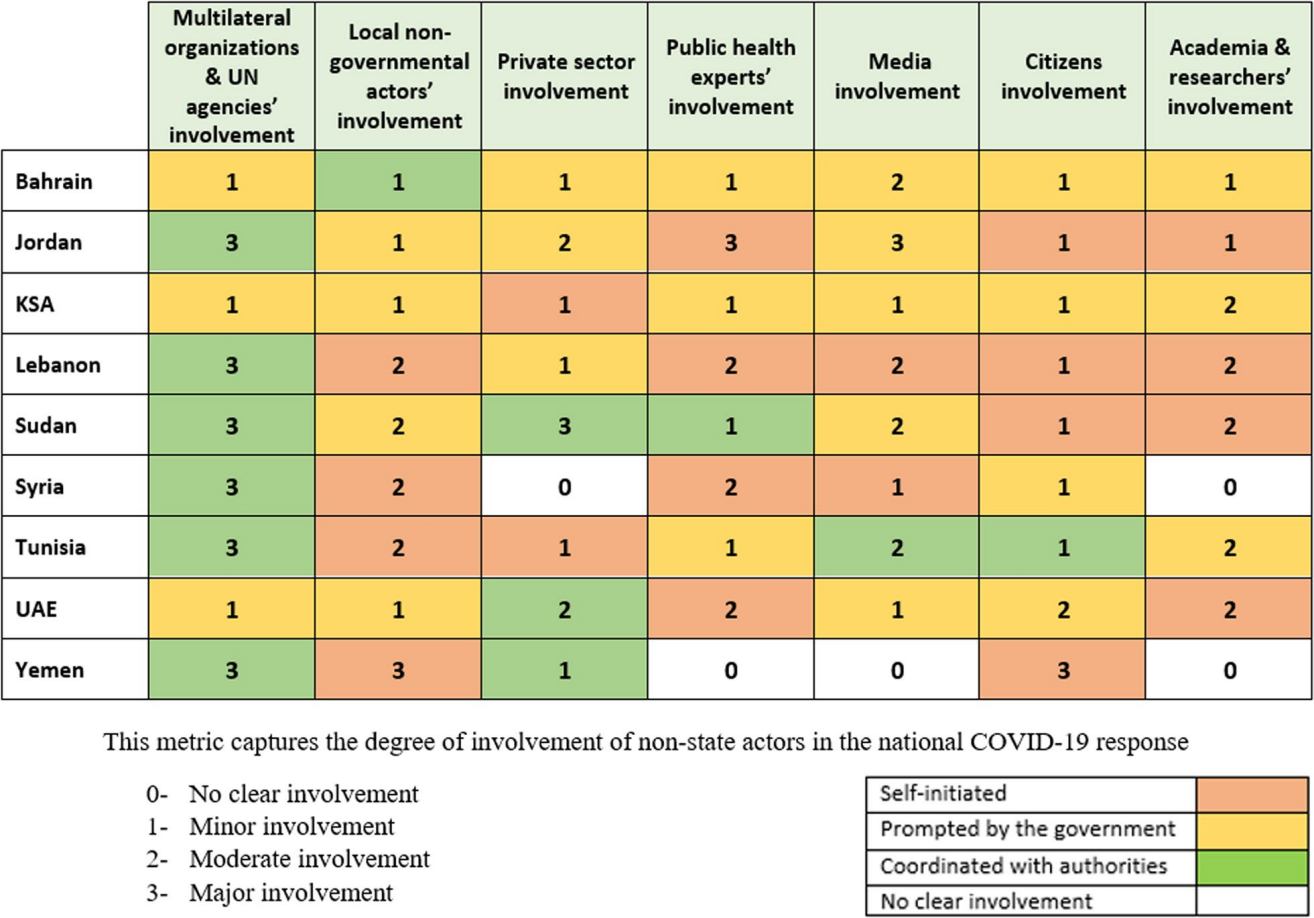
Involvement of non-state actors and their roles in the COVID-19 pandemic response

The involvement of intergovernmental organizations (e.g. WHO, UN entities) and CSOs was more prominent and proactive in low-income countries (e.g. Sudan, Syria, Yemen) and countries with a high number of refugees (e.g. Jordan, Lebanon) where they already had a strong presence prior to the pandemic. In these settings, non-state actors leveraged their existing presence and expertise to complement government initiatives and address gaps in awareness-raising and community outreach. In contrast, non-state actors’ involvement in high-income countries were largely prompted by the government [82–85] (Fig. 1). The United Arab Emirates provides a notable example of private sector engagement in the pandemic response. For instance, the country launched the first United Nations Alliance for Disaster Resilient Societies (ARISE) initiative in the region, coordinating and maximizing private sector involvement in disaster management [86]. Additionally, government and private sector entities collaborated to support residents’ mental health needs during the crisis by providing access to free support services, such as hotlines, webinars and counselling [86].

While COVID-19 has increased the need for experts in various public health disciplines to plan suitable programs and responses, the selected countries of the EMR have registered only modest initiatives from public health experts, whose engagement has not been systematically integrated. Similarly, the role of researchers and academia has not been well integrated into the national pandemic responses. Only in three of the countries studied (KSA, UAE and Tunisia) did the government allocate funds for COVID-19 research as part of the national response (Fig. 1).

### Administration, funding and evaluation

Every collaborative needs effective administrative practices, adequate financial resources and a plan for regular monitoring and evaluation [7]. Below, we elaborate on each of these elements with respect to the multi-sectoral response in the selected countries.

#### Administration of the multi-sectoral response

In the majority of the selected high- and middle-income countries of the EMR, the organization of the COVID-19 response was highly centralized, owing largely to the centralization of health services. Those countries with centralized governance and strong leadership, such as Jordan, KSA, Bahrain and the UAE, were able to swiftly coordinate and mobilize resources across various sectors. Conversely, in countries with fragmented governance or weak leadership, such as Syria, Sudan and Yemen, multi-sectoral collaboration was often hindered by conflicting priorities, the emergence of multiple power centres and a lack of clear direction.

While a centralized approach may be logical in addressing a crisis of such scale, it is also crucial to harmonize and integrate the top-down approach with local-level actions and implementation. This emphasizes the importance of considering local context and involving communities in the implementation of policy intervention. Poor governance coherence has been attributed to factors including a lack of legitimacy of authority and trust in leadership, and the absence of clear mechanisms to translate high-level policies and regulations into practical measures that can be implemented, enforced and monitored at the local level.

#### Financial resources

The amount and source of funding dedicated to the national response differed across the nine selected countries. Unsurprisingly, governments in high-income countries (Bahrain, KSA and UAE) invested the highest amounts [87–89]. Next were upper-middle-income countries (Jordan, Lebanon and Tunisia) where, in addition to government’s dedicated funds and foreign support, several platforms were established to raise funds from public donations to support the response. In contrast, low-income countries (Sudan, Syria and Yemen) were reported to have insufficient financial resources to develop policies and engage multiple sectors in implementation activities. Consequently, these countries tended to heavily rely on external funders and donors (Table 5). With the exceptions of the UAE and Jordan, none of the selected countries offered clear information on the cost-sharing mechanisms between sectors for specific components of the plan.

**Table 5:** Overview of funding, monitoring and evaluation and accountability mechanisms in selected countries

Country	Sources of funding for implementation of the response	Financing mechanisms/arrangement in place	Mechanism for monitoring and evaluation (responsible entity)	Accountability framework
Bahrain	Government	Not found	Yes (Bahrain National Task force)	Not found
Jordan	Government; Local and foreign donations	Yes	Not found	Not found
KSA	Government	Not found	Not found	Not found
Lebanon	Government; local and foreign donations	Not found	Yes (Ministry of Public health)	Not found
Sudan	External donors; private sector; government	Not found	Yes (COVID-19 Working Group with support from Information Management Working Group)	Not found
Syria	External donors; government	Not found	No formal process (UN Country Team and WHO)	Not found
Tunisia	Government; local and foreign donations	Not found	Yes (National COVID-19 Monitoring Authority)	Not found
UAE	Government	Yes	Yes (National Emergency Crisis and Disaster Management Authority, NCEMA)	Not found
Yemen	External donors; government	Not found	No formal process (various multilateral organizations and existing emergency operations centres, EOC)	Not found

Across the nine selected countries, it was not clear to what extent budgeting for activities of the collaborative was a multi-sectoral activity, undertaken by all sectors (and stakeholders) involved. And while the budget covered the policy interventions under the response plan, there was no explicit reference to the expenses incurred to run the coordination mechanisms at national and subnational levels.

#### Monitoring and evaluation

Collaborative progress needs to be monitored through an accountability framework consisting of indicators related to both process and outcomes, pegged to the objectives of the joint action plan. With the exceptions of Syria and Yemen, most countries had mechanisms in place and designated entities for reporting on progress; however, only a few countries incorporated explicit indicators for monitoring and evaluation into their national response plans (Table 5). Furthermore, none of the countries included indicators to monitor the process of the collaborative itself. Information on the mechanism and designated entity for monitoring and evaluation were not identified for Jordan and KSA. In all cases, it was unclear to what extent monitoring and evaluation were formally being conducted on the ground.

While accountability is an essential governance element in collaboratives, critical for building trust and enhancing effectiveness, none of the selected countries incorporated an explicit accountability framework or integrated anti-corruption and counter-fraud measures into their multi-sectoral plans or coordination mechanisms. A recent report conducted in the region highlighted corruption risks in public procurement and privately donated funds, and urged governments to adopt transparency and accountability measures to provide much-needed anti-corruption oversight [90].

### Degree of multi-sectoral engagement

Multi-sectoral engagement lies on a spectrum, from more passive to active involvement. It can range from communication, in which information from one sector is shared with other sectors; to cooperation, which involves optimizing resources while establishing formalities in the work relationship; to coordination, in which there is increased horizontal networking among sectors with some sharing of financing sources; and to integration, which entails systematic integration of objectives and administrative processes and the sharing of resources, responsibilities and actions [13].

Across the selected EMR countries (and based on the findings from the documentation review and stakeholders’ inputs), the degree of multi-sectoral engagement between sectors and actors spanned the spectrum from communication to cooperation and coordination; it rarely went further to integration, which necessitates formal partnerships and shared policies and practices to ensure achievement of a common goal. Although low-income countries witnessed the engagement of sectors outside the health sector, this engagement seemed mostly limited to providing information or viewpoints and was not a truly collaborative effort. Across middle-income countries, the engagement could be classified as cooperation, involving formal meetings and regular exchange of staff, information and practices. As for high-income countries, the engagement went further, to coordination with sharing on regular formal bases and regular exchanges and specific undertaking on shared projects. Table 6 provides a visual representation of the degree of multi-sectoral engagement across the selected countries.

**Table 6:** Degree of multi-sectoral engagement across selected countries

	Co-existence	Communication	Cooperation	Coordination	Collaboration
Bahrain				X	
KSA				X	
Jordan			X		
Lebanon			X		
Sudan		X			
Syria		X			
Tunisia			X		
UAE				X	
Yemen		X			

### Impact

Strong multi-sectoral collaboration has been shown to yield a more effective pandemic response [1, 91]. Across the countries selected, those with a stronger degree of multi-sectoral engagement (see Table 6) seem to adopt a more comprehensive set of COVID-19 measures or interventions and achieve better COVID-19-related outcomes (see section Comprehensiveness of policy measures and responses and Box 1, respectively). However, it is important to caution that this study does not claim any cause–effect relationship. Other factors may also have contributed to the observed variations, including population demographics, prevailing political structures, disparities in health system capacities and cultural factors ranging from high levels of socialization to distrust in public institutions, which might influence compliance with public health measures.

**Table:** 

**Box 1: Case Study on Active Surveillance and Contact Tracing in the selected EMR Countries** The varied approaches employed in active surveillance and contact tracing – a cornerstone of public health responses – exemplify the diverse challenges and capacities among the selected EMR countries in managing public health crises such as the COVID-19 pandemic • In high-income countries such as Bahrain, Saudi Arabia and the United Arab Emirates, robust digital surveillance is exemplified through the health electronic surveillance network, which significantly aided in disease detection, response and community health monitoring [5, 6, 103, 104]. The “Tawakkalna” application, developed by the government in Saudi Arabia, played a pivotal role in tracking infections, saving lives and reducing the strain on health facilities [5, 6]. Similarly, Bahrain introduced the BeAware Bahrain app, leveraging cloud technologies and location tracking to identify and alert individuals in contact with COVID-19 cases [104]. The United Arab Emirates implemented a comprehensive strategy focusing on mass testing, contact tracing and isolating positive cases. Early adoption of this strategy in April 2020 contributed to a lower-than-expected mortality rate [103] • Middle-income countries (Lebanon, Jordan and Tunisia) faced challenges due to limited capacity in detecting, tracing and isolating COVID-19 cases [45, 105, 106]. Factors such as under-resourced surveillance systems, delayed diagnostic scale-up, inadequate isolation facilities and the absence of digital contact tracing contributed to underestimation of community spread and hindered informed decision-making. Lebanon, for instance, heavily relied on a single hospital for testing, isolating and treating confirmed cases during the early stages of the pandemic, which undermined the overall response [45] • Low-income countries such as Sudan, Syria, and Yemen faced significant challenges attributed to security concerns and limited accessibility [107–109]. The absence of a unified, digitalized health information system hindered the tracking of the pandemic’s evolution and response. Sudan, for instance, grappled with a shortage of isolation centres and testing capacity, underscoring the broader difficulties in managing the pandemic in such contexts [108]	

#### Comprehensiveness of policy measures and responses

**Fig. 2:**
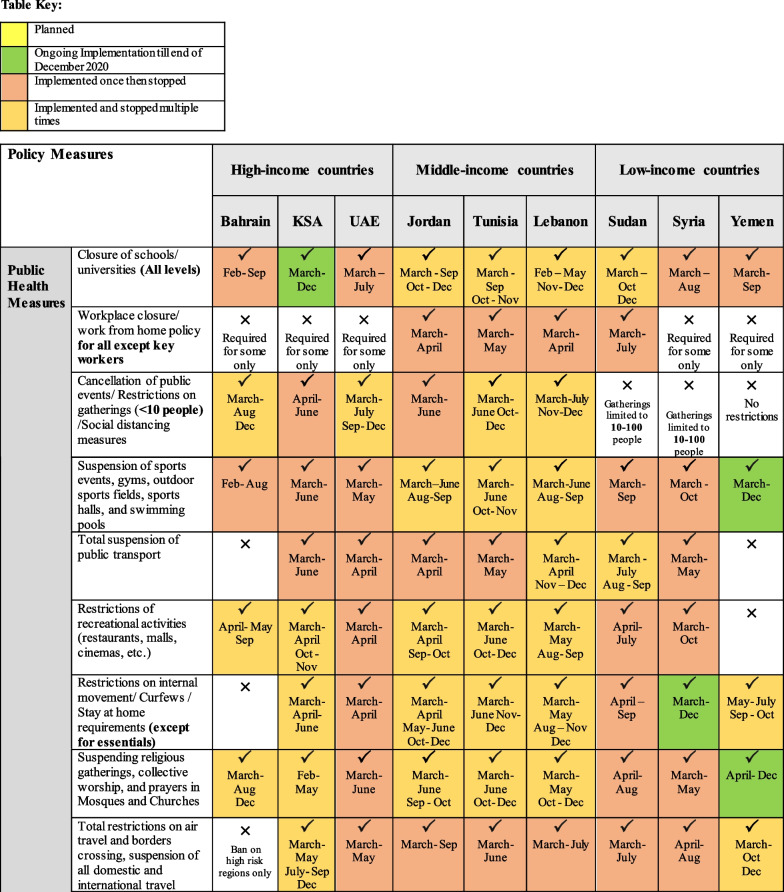

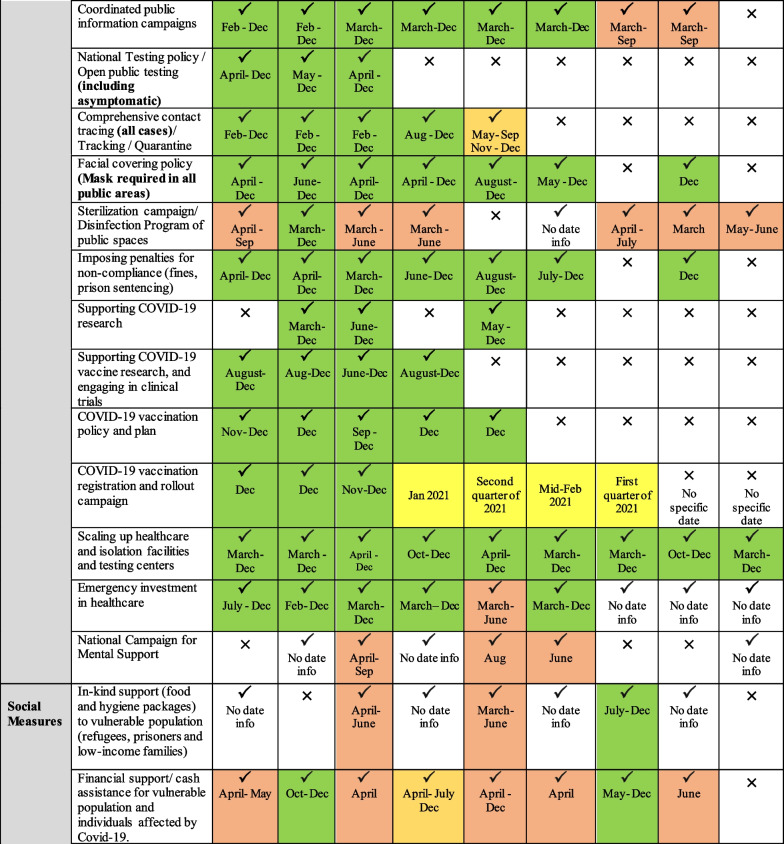

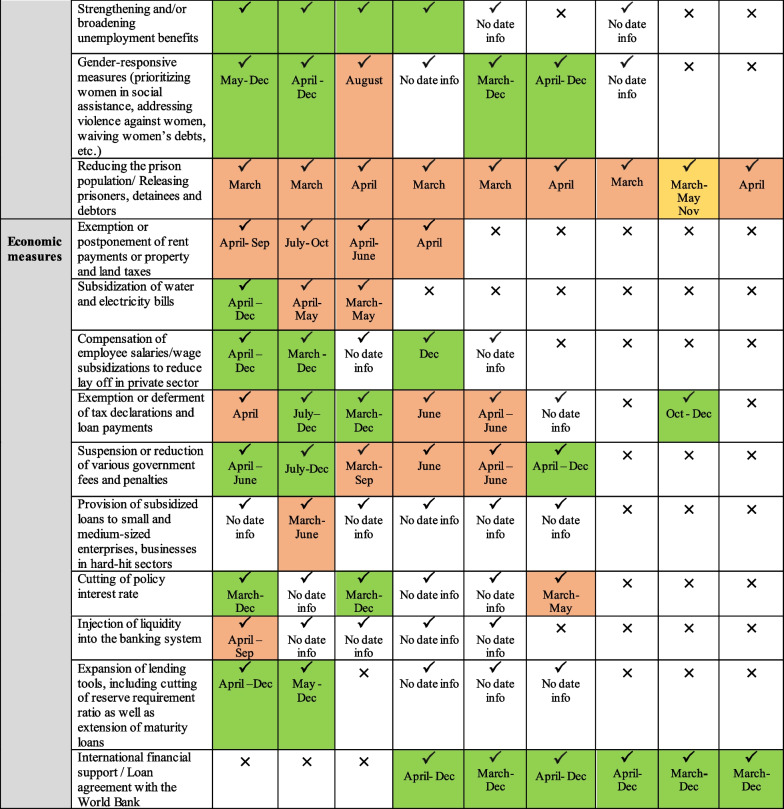
Overview of public health and socio-economic measures adopted by selected EMR countries during the first year of the pandemic (i.e. year 2020)

Governments in the EMR have responded differently to the pandemic, with variations in reaction speed and strictness of implementation. Collectively, country responses ranged from public health measures such as lockdowns, social distancing and contact tracing to social interventions and broader fiscal policies to restore the economy. These responses continued to change over time as countries experienced second waves of outbreaks or recovered from major bouts of infection. While all selected countries implemented a range of public health measures, variations across income groups were more pronounced in terms of the comprehensiveness of adopted social and economic measures (Fig. 2).

Across income groups, policy measures were most comprehensive in high-income countries, followed by middle-income countries, with the least number of measures adopted in low-income countries. Box 1 illustrates the varied approaches employed in active surveillance and contact tracing among the selected countries in the EMR.

The overall pandemic response in the EMR can be grouped into three broad types:

#### Early response, proper measures and successful implementation: KSA, UAE and Bahrain

KSA began taking precautionary actions early in January even before WHO declared COVID-19 a pandemic. The whole-of-government approach adopted in KSA at the national level allowed the right decisions to be taken on time and to be implemented promptly and in a coordinated manner. The UAE was the first country in the EMR to report a COVID-19 case; in late January 2020. The UAE had a clear strategy that relied on communication and coordination, with no room for rivalry, opposition or dissonance; this has allowed the country to rise to the forefront of effective decision-making. In fact, it ranked among the world’s top 10 for COVID-19 treatment efficiency and among the world’s top 20 for the implementation of COVID-19 safety measures [92]. Similarly, the WHO’s Eastern Mediterranean office commended Bahrain’s swift and effective countermeasures against COVID-19 as an example that other countries should follow [93, 94].

#### Successful handling of the first wave, but lost control later: Jordan, Lebanon and Tunisia

Jordan’s response to the pandemic was one of the strictest in the region and among the world in the first months of the outbreak. Later, after easing lockdown measures, Jordan witnessed a gradual increase in confirmed cases of COVID-19 by early August 2020. Similarly, Tunisia and Lebanon’s initial response highlighted their inability to manage a crisis of such severity. Tunisia managed to successfully control the initial epidemiological threat posed by the pandemic, with the second-lowest fatality rate in the region [95]. However, the government was not as proactive as it could have been, effectively implementing needed measures almost two weeks after the confirmation of the first COVID-19 case. As for Lebanon, at the start of the pandemic the government was successful in handling the response; however, after the Beirut port explosions on 4 August 2020, the response got out of control and cases began to skyrocket [47, 96, 97]. This has been attributed to the failure of the government to develop a long-term strategy and the breakdown of communication across sectors.

#### Failed response due to crippling war: Yemen, Syria and Sudan

The pandemic has hit Sudan, Syria and Yemen at a critical time, leaving the authorities in each of these countries struggling to deal with the newly imposed public health threat. Yemen was one of the last countries worldwide to announce the first confirmed case of COVID-19 infection among its population, and 1 year after the start of the pandemic, Yemen had the lowest publicly reported infection rate in the region and the highest case fatality rate in not only the region but the world [98, 99]. Syria had no official number of COVID-19 tests conducted until 16 March 2020, with only 103 tests reported in government-held areas. Despite the low reporting, many indicators suggested that Syria already had a considerable number of COVID-19 cases prior to the announcement of the first official case on 22 March 2020 [100–102]. The lack of transparency and weak reporting system in these three countries has affected the national COVID-19 response and led to confusion and mistrust at different levels of decision-making, undermining multi-sectoral action.

#### Selected COVID-19-related outcomes

**Fig. 3:**
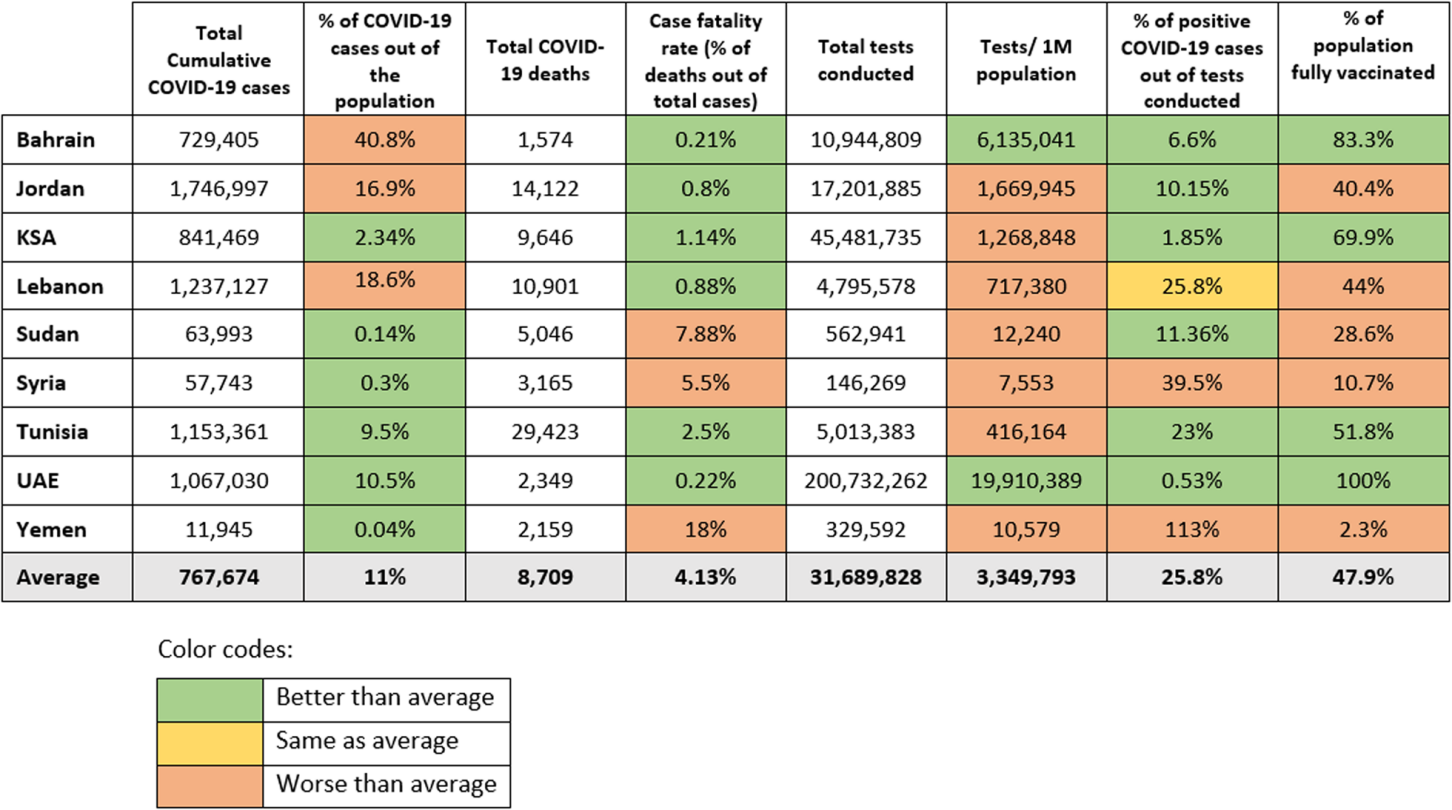
Covid-19-related outcomes in selected EMR countries (as of 5 May 2023) [74–76, 111–113]

As of 5 May 2023, when the WHO declared an end to the COVID-19 pandemic as a public health emergency [110], a cumulative total of 7 million confirmed severe acute respiratory syndrome coronavirus 2 (SARS-CoV-2) infections and 78,000 COVID-19 deaths had been reported across the nine selected countries of the EMR. By the end of the pandemic, Bahrain had the highest percentage of COVID-19 cases out of the population (40.8%) followed by Lebanon (18.6%), then Jordan (16.9%). Yemen had the highest case fatality rate (CFR) at 18%, followed by Sudan (7.9%), then Syria (5.5%). In contrast, UAE and Bahrain registered the lowest CFRs, both at 0.2% (Fig. 3).

UAE and Bahrain also conducted the highest number of tests per million population, while Syria conducted the fewest. As for the percentage of positive COVID-19 cases out of the total tests conducted, Yemen had the highest percentage (113%) followed by Syria (39.5%), Lebanon (25.8%) and Tunisia (23%). The WHO recommends a positivity rate of around 3–12% as a general benchmark indicating adequate testing [108]. A high positivity rate indicates that the country is not testing widely enough to find all cases, and the actual number of circulating cases in the community is far higher than the confirmed numbers.

Regarding vaccination, UAE had the highest percentage of its population fully vaccinated (100%), followed by Bahrain (83.3%) and KSA (69.9%). Yemen, Syria and Sudan had the lowest vaccination rates, with 2.3%, 10.7% and 28.6%, respectively (Fig. 3).

It is important to account for the inconsistencies in methodologies used to report cases among countries due to internal and external factors. For instance, given the limited testing activity and capacity across Syria, Sudan and Yemen, and the lack of credible information sharing and transparency, the actual number of cases likely exceeds available official figures.

### Enabling factors

#### Key reported barriers

Barriers to multi-sectoral action were more acute in the fragile countries selected for this study (Syria, Sudan and Yemen) and to a lesser extent in middle-income countries (Lebanon, Jordan and Tunisia) where weak institutions, sectoral bureaucracy and fragmentations have undermined coordination. This is in contrast to high-income countries (KSA, UAE and Bahrain) where health system infrastructures are more advanced, robust multi-sectoral structures existed prior to the pandemic and resources were available to quickly activate the existing multi-sectoral structures or establish new ones as part of the response to the newly imposed public health threat.

One of the most recurrent barriers was the fragmentation of authorities and the multiplicity of actors in charge of the pandemic response, which created confusion and undermined public trust in the pandemic response. This was more pronounced in conflict-affected countries of the EMR where national governments often suffered from perceived lack of legitimacy by opposing parties internally as well as by other governments and international actors. Key informants raised these concerns and further highlighted the lack of a mandate defining the roles and responsibilities of each sector, as well as a lack of clear ownership. Additionally, amidst the lack of mechanisms for conflict management and building trust as part of the national pandemic response, key recurrent challenges included competition over resources and conflicts between sectors and actors at different levels, especially in fragile countries where political consideration was prioritized over the health of the population. In many cases, political, personal and financial interests were reported to be at the core of these conflicts, which hindered the willingness of different actors to carry out and sustain inter-sectoral action.

Siloed thinking and resistance to adopting multi-sectoral perspectives were also highlighted as challenges that apply to the health sector, as well as to other sectors. This is mainly attributed to the limited understanding of the importance of multi-sectoral collaboration and how best to promote and support multi-sectoral action for health at the national level. As re-iterated by participants, multi-sectoral action is still a new concept for the majority of governments in the EMR, where working in silos is still the norm. This is also related to the limited awareness about the wider determinants of health and that the responsibility of responding to health problems lies beyond the mandates of the Ministry of Health.

Another main concern raised was the lack of processes for accountability and monitoring. In fact, 1 year after the start of the pandemic, some countries in the EMR were still not making their COVID-19 data public, or were not sharing the information, models and assumptions upon which decisions were made. Consequently, this ‘perceived secrecy’ has undermined public trust in many leaders and negatively affected the goal of reaching a successful multi-sectoral response. The implementation of accountability mechanisms and monitoring frameworks was seen to be difficult and sometimes impossible, especially in countries where entrenched political and administrative corruption was reported. A lack of accountability within the same department or ministry was also reported, and not only across the different ministries involved in the response (i.e. lack of vertical and horizontal reporting and accountability). This further hindered the effective translation and implementation of guidelines and high-level recommendations into practice at the local levels.

Participants noted that coordination at the beginning of the pandemic was more robust as compared with 1 year later, hence failure to sustain the multi-sectoral response for coordinated efforts was one of the main challenges experienced. As highlighted by some participants, it was difficult to maintain the active involvement of all sectors, state and non-state actors in implementing the policy response over a long period of time, as the perceived threat started to decrease as well as the available funds, with economic considerations taking precedence over public health concerns. In fact, it was noted by several participants that the inadequate finances and the over-reliance on external donors to support implementation capacity meant that policies and responses were not being implemented in a sustainable way. Furthermore, inadequate anchoring of the multi-sectoral governance structure within government in some of the selected countries undermined their convening power and ability to secure a budget line item.

The prevailing political context has been another reported factor influencing multi-sectoral collaboration. In Lebanon, the unprecedented economic crises and the more recent Beirut port explosion shifted political commitment away from the pandemic, weakening the collaborative and the overall response. In Yemen, the COVID-19 crisis has become yet another politicized element of the ongoing conflict, a way for the opposing parties to point at each other’s failures or even accuse the other of helping spread the virus.

#### Key reported facilitators

Despite the challenges faced, it is important to note that as a result of the pandemic, progress has been perceived by study participants in the area of multi-sectoral collaboration in the selected countries. When it comes to facilitators, prior relations between sectors and experiences from dealing with previous emerging infectious diseases have been reported. For instance, in the UAE, the pandemic response management was centralized through the pre-existing National Emergency Crisis and Disaster Management Authority (NCEMA), established in 2007. Similarly, the robust preparedness and public health response capabilities in KSA were strengthened by the valuable experience gained from previously managing the Middle East respiratory syndrome coronavirus (MERS-CoV) in 2012 and from decades of planning religious mass gatherings in the face of numerous public health challenges.

Participants also acknowledged the scale and breadth of the COVID-19 pandemic and the urgent need to mitigate its devastating impact, facilitate political commitment and recognize interdependencies over a common threat.

## Discussion

Mirroring global efforts, this study demonstrates that the selected nine countries in the EMR are making efforts to incorporate multi-sectoral action into their pandemic responses.

Despite these efforts, persistent challenges and gaps remain, presenting untapped opportunities that can be leveraged for more efficient public health responses in the future. Barriers to multi-sectoral responses were more acute in fragile countries and to a lesser extent in middle-income countries where weak institutions, sectoral bureaucracy and fragmentations have undermined coordination. The most frequently reported barriers across the selected EMR countries were poor multi-sectoral culture; fragmentation of authorities and multiplicity of actors in charge; lack of ownership and mandates specifying roles and responsibilities of different sectors and actors during public health emergencies; competition for resources and lack of mechanisms for conflict management; weak legitimacy of authorities and distrust in government leadership and public institutions; absence of a process to effectively monitor and evaluate the impact of adopted measures and the overall collaborative arrangement; limited resources and funding available for implementing the multi-sectoral response exacerbated by the lack of sustainable joint financing mechanisms; difficulty in sustaining priority for collaboration; and the politicization of the pandemic.

As health challenges increase in complexity, multi-level and multi-disciplinary public health interventions will become the norm [114]. In this regard, it will become increasingly important to capitalize on existing governance functions, institutional structures, mechanisms and partnerships, as well as on research and data ecosystems to reduce duplication of efforts and waste of resources [114]. Governments must continue to build upon the lessons learned from the COVID-19 pandemic to improve and institutionalize multi-sectoral efforts and better be prepared to respond to future crises [91]. Below, we put forward recommendations for strengthening multi-sectoral collaborations for public health emergency responses in the EMR. We also incorporate wider considerations on how the current COVID-19 responses can be used as a window of opportunity to build greater resilience in health systems in the region. The recommendations are grouped into three broad categories: governance and leadership functions; institutional structures, processes and mechanisms; and research and data ecosystems.

At the governance and leadership level, it is critical to ensure a shared understanding and alignment of interests across sectors and actors. Defining a shared vision and agreeing on specific goals are essential for effective and impactful multi-sectoral collaboration [115]. The approach to leadership during public health crises can vary on the basis of the context, governance structures and public health systems of different countries. Centralization and decentralization within and between governments can change in a pandemic; the politics of credit and blame shape politicians’ approaches to problems in complex and context-dependent ways [116]. While there is no one-size-fits-all approach, countries could opt for a hybrid approach, where there is a central coordinating body or leader overseeing the response, but with active involvement and coordination across different sectors at various levels (local, regional and national).

To further enhance collaborative sustainability, leadership capacity needs to be developed across sectors and levels of government, and champions fostered in different sectors that agree on common objectives [117]. Strong coordination is needed to ensure that all sectors are working collaboratively to achieve a whole-of-government, whole-of-society response for addressing national challenges and building resilient health systems [118]. This, in turn, requires a discrete set of governance structures as well as stewardship and leadership skills that broaden collaboration in both horizontal and vertical dimensions [119]. Importantly, there is a need to mobilize and allocate adequate resources for engaging multiple sectors to execute the mandate of the collaboration. Budgeting for collaborative activities should be a joint, multi-sectoral activity [12], and funds should be earmarked (whether through multi-sectoral co-financing, joint funding or new financing solutions) to guarantee the execution of the mandate [120].

At the level of institutional structures, processes and mechanisms, there is a need to create a central coordinating structure that includes representatives from various sectors and actors. While having a multi-sectoral committee is a good start, it does not guarantee success [115]; permanent multi-sectoral structures and mechanisms are preferred for their improved chance of sustainability and longevity; and increased effort should be channelled towards the dynamics of the structure: its mandate, clarity of goals and clarity of roles [8, 115]. Additionally, for collaboration to function effectively, the structure needs to have authority to make decisions and coordinate actions across sectors as well as legitimacy to hold others accountable and manage power conflicts [7]. Importantly, well-implemented communication channels and community engagement processes can support the design of context-specific interventions, build trust in public institutions and provide logistical and administrative support during public health crises [121]. Measurement and evaluation systems can also serve as powerful tools for governing multi-sectoral action [122]. This requires shared indicators that monitor the health, economic and social impacts of the multi-sectoral action, as well as the internal processes and procedures of the collaborative activity [123]. Gathering these data necessitate a shared measurement system involving standardized and ongoing data collection across sectors and actors [123, 124]. There is also a need to reinforce the value of multi-sectoral collaboration, with a focus on competencies for implementing cross-sectoral initiatives.

At the level of research and data ecosystems, governments should strengthen and institutionalize the use of research evidence and public health expertise in decision-making processes. The establishment of a strong evidence base is critical to complement and supplement good governance, fight infodemics and promote trust and accountability [12]. The COVID-19 pandemic can also serve as an impetus to address long-standing underinvestment and undervaluation in health information systems and routine sources of data [125]. Timely data from information and surveillance systems not only informs outbreak response but can also generate much-needed evidence to strengthen the overall health system resilience. Having reliable data can also help set indicators and targets that can be monitored across the collaborative. Finally, it is important to establish transparent mechanisms for coordinating and integrating research, data and expertise across sectors that can enable more effective, efficient and swift responses to increasingly complex public health challenges facing the EMR and the world [77].

### Limitations

A limitation of this study is the inclusion of only nine countries from the region; the findings may thus not be generalizable to all EMR context. It is important to scale up the study to other countries in the region. Additionally, while 24 purposively selected stakeholders were initially invited to participate in the key informant interviews, only 8 responded. Nonetheless, these stakeholders represented different sectors including governments, healthcare organizations, non-governmental organizations and academia, which enabled diverse perspectives. In addition, data triangulation helped increase the reliability and validity of findings through cross-checking of information across different data sources. Finally, despite our attempt to conduct a comprehensive search of the literature, we may have missed out on unpublished and not easily accessible data. This is unlikely to significantly change our results.

## Conclusions

The COVID-19 pandemic has triggered unprecedented efforts towards multi-sectoral action. Mirroring global efforts, this study demonstrates that the selected nine countries in the EMR are making efforts to integrate multi-sectoral action into their pandemic responses. Nevertheless, persistent challenges and gaps remain, presenting untapped opportunities that governments in the selected EMR countries can leverage to enhance the efficiency of future public health emergency responses. The methodology and framework developed for this study can be replicated in other settings to assess multi-sectoral collaborations and initiatives to respond to public health crises. Study findings can inform multi-sectoral initiatives’ improvement efforts in the EMR and beyond, with a particular emphasis on fragile settings.

## Data Availability

All datasets are presented in the main manuscript.
